# Implications of providing social support to close network members on the social well-being of older people in Kitui County, Kenya

**DOI:** 10.3389/fragi.2022.897508

**Published:** 2022-11-16

**Authors:** Kezia Mbuthia, Samuel Mwangi, George Owino

**Affiliations:** Department of Sociology, Gender and Development Studies, Kenyatta University, Nairobi, Kenya

**Keywords:** social well-being, social support, instrumental support, emotional support, informational support, close network members

## Abstract

Social support is a strong predictor of social well-being. Older people are key providers of social support to others, but an overemphasis on received social support in gerontological literature portrays them as mere recipients. We examined the association between social support provided by older people and its influence on their social well-being. Data were collected from 369 respondents residing in rural dwellings in Kitui County using mixed methods and were analyzed for association using chi-square statistics. Instrumental, emotional, and information support provision was determined by asking four questions in each category about whether the respondents provided social support to close network members. The subjective experience of support provision using a satisfaction question for each domain of social support was used to determine the influence of providing social support on the social well-being of older people. Provision of social support across the three domains was found to be significantly associated with social well-being. The level of statistical significance was highest for emotional and information support compared to instrumental support. Older people are important providers of social support. The majority of those who provided social support reported being satisfied. Therefore, offering social support, especially emotional and information support, is an important contributor to satisfaction with these aspects of social well-being.

## 1 Introduction

Social well-being can be defined as a person’s evaluation of their situation, social relations, and how well they can function in a community (Dunaeva, 2018). This construct has been classified into two dimensions: subjective experiences and objective circumstances (Boreham, Povey, & Tomaszewski, 2013). Objective circumstances relate to support that is apparent, tangible, and direct (Yu et al., 2020).

Subjective experiences reflect a person’s internal thoughts and feelings that are not visible to outside observers (Lucas, 2018). A singular objective life situation or circumstance can be evaluated differently by different people. The subjective experiences are evaluations of life in terms of satisfaction (Keyes, Shmotkin, & Ryff, 2002) based on objective circumstances. According to Sen, Prybutok, and Prybutok (2021) social well-being involves both external and internal factors, such as the presence of exchanges or connections and satisfaction with the quality of those exchanges or connections. Therefore, in the context of this study, social well-being refers to satisfaction with the quality of social support provided. However, although the social well-being of older people is a topic that has drawn scholarly consideration in high-income nations, it has received little attention in low- and middle-income countries (Elliott, 2017).

According to Dunaeva (2018), social support is a significant element of social well-being. It relates to social exchanges that are regarded as beneficial (Dykstra, 2015), such as advice, encouragement, and love (Thoits, 2011). These supportive qualities sustain social relationships (Umberson & Montez, 2010). From this definition, similar to Schwarzer, Knoll, and Rieckmann (2004), social support is investigated in several types such as emotional (i.e., love), informational (i.e., giving advice), and instrumental (i.e., assistance with a problem). It serves as a potential resource to individuals and is associated with well-being benefits, especially among older adults (Mohd et al., 2019).

Social support is described as either perceived or received support. Received social support refers to the actual reported exchanges, while perceived social support refers to potential access to support by an individual (Haber et al., 2007; Uchino, 2009). The multidimensionality of social support has led to some of its aspects being given more attention than others, which may be attributed to the lack of a standardized method of measuring and reporting the multiple dimensions (Mohd et al., 2019). Specifically, perceived social support has attracted wider scholarly attention than received social support (Mohd et al., 2019).

Received social support is said to be provided and received in social relationships. Received social support has attracted wide theoretical and empirical attention, unlike provided social support (Song, Son, and Lin, 2011). This is especially the case when investigating social support among older people. Received social support has been found useful in providing protection against threats to well-being and in helping people to deal with setbacks that are detrimental to social well-being (Dykstra, 2015). The overemphasis in gerontological literature of social support received by older people portrays them as mere recipients and disregards their role as its providers (Albertini, Kohli, & Vogel, 2007; Dykstra, 2015). Some studies found that provided social support was beneficial to providers. For instance, Bai et al. (2020) observed that providing social support enhanced well-being in older people who took care of their grandchildren compared to people who did not. Other studies however, found that providing social support elicits feelings of burden and frustration (Thomas, 2010; Morelli et al., 2015) and is overwhelming (Krause & Shaw, 2002) for older people. The mixed and contradictory findings call for more research to understand the influence of providing social support on well-being. Numerous crucial aspects of social support provision and its effects on social well-being have not been systematically documented (Thomas, 2010). This is because prior works focus on two categories of social support, instrumental, and emotional, with each category affecting support providers differently (Morelli et al., 2015). Therefore, this study focused on three categories of social support, instrumental, emotional, and information, to assess their effect on support providers because the findings in prior studies are mixed and contradictory.

In addition, studies on social support infrequently examine whether helping others has any benefits. According to Brown et al. (2003), the effects are frequently attributed to receiving support or occasionally to reciprocal support. Even if some social support measures actually appear to examine giving, they do so possibly inadvertently. Therefore, the study aims to investigate the effects of providing instrumental, emotional, and informational social support to others on the social well-being of the providers (i.e., older people).

## 2 Materials and methods

### 2.1 Participants

A cross-sectional survey design was employed to collect primary data in June and July 2021. The total study population of older people in four sampled sub-counties of Kitui County was 32,839, according to the Kenya National Bureau of Statistics (KNBS, 2019). Using Yamane’s (1967) formula at a 95% confidence level and *p* = 0.05, a sample of 396 older people was determined. Multistage cluster sampling was employed, and participants within each of the clusters were selected using a simple random sampling technique.

In the first stage, administrative locations in the four sub-counties were identified. In each sub-county, three locations were selected using the fish bowl draw method (Kumar, 2011). In the second stage, two sub-locations were purposively identified from the selected locations, and a sampling frame (i.e., list of older people living within the sub-locations) was obtained from the area administrative chiefs and village elders. These steps were necessary because the population was geographically heterogeneous (Sedgwick, 2015), and the sampling units for the different stages were different (Acharya et al., 2013). The names of older people provided by the area chiefs and village elders were entered into Microsoft Excel 2016. Older people in the sampled sub-locations were randomly selected using the Rand function.

### 2.2 Measures

#### 2.2.1 Independent variable: Social support

Participants were asked to report whether they provided social support to close network members (CNMs), including spouse, children, neighbors, relatives, and friends, in the 12 months prior to the study. The study measured three domains of social support with four items in each: (a) instrumental social support—lend or give money, help with chores/errands, provided care in sickness, lent items or tools; (b) emotional support—cheered up or helped CNM feel better, showed interest in their personal life, did or said things that were kind or considerate, trusted CNM to solve their problems; (c) informational support—gave helpful advice when needed to make important decision, agreed with CNM’s actions or thoughts, gave information to understand an issue, and gave CNM feedback on an action they wanted to take. These items in the three domains of social support were drawn from the previous studies (Morelli, Lee, Arnn & Zaki, 2015; Schwarzer et al., 2004; Thoits, 2011; Newsom et al., 2005). Participant’s response categories were: Yes [1] or No [2].

#### 2.2.2 Dependent variable: Social well-being

The study measured social well-being by assessing how the respondents appraised the social support that they provided using a satisfaction question for each domain. The satisfaction question was indexed by responses (from 1 = extremely dissatisfied, 2 = dissatisfied, 3 = satisfied, and 4 = extremely satisfied) to the following statement: In the last 12 months, how satisfied are you with the social support that you provided to CNMs? The responses were highly skewed because very few respondents selected extremely dissatisfied and extremely satisfied in all the three domains of social support. At the analysis stage, responses for 1 and 2 were collapsed from the original scale to 1 = dissatisfied and those for 3 and 4 were collapsed to 2 = satisfied, creating a dichotomized scale. The research instrument was pre-tested with 30 respondents from a subsample of older people, and a post-test was conducted 3 weeks later. The test–retest reliability was reasonably measured by the correlation coefficient between the two scores, which was 0.718.

### 2.3 Procedure

A questionnaire was developed for this study that covered the three domains of social support and the social well-being question, as well as socio-demographic questions, including gender, participant’s marital status, level of education attainment, source of livelihood, and average monthly income. These were administered by the researcher and the research assistants at the respondent’s homes. Four focus group discussions comprising older men and women were also conducted, and the data were used to complement the quantitative data from the questionnaire.

With the assistance of local leaders, a distinct group of older people from those who filled out the questionnaire were purposively sampled. This was done in order to include individuals who could stimulate one another to explore their opinions and experiences in light of the discussion topics (Moser & Korstjens, 2018). The focus group discussion themes were developed using the quantitative data from the questionnaire. For instance, the questionnaire was divided into five sections: demographic characteristics of the respondents, instrumental, emotional, and informational social support, and each domain’s satisfaction questions. These social support domain questions served as the basis for the focus group template and were the starting point for addressing the crucial concerns of “why” and “how” providing social support influenced satisfaction with doing so. This was done in order to understand the reasoning underlying participants’ feelings and actions. The discussants were welcomed to the FGD, and the purpose of the study was explained to them. The researcher moderated the focus group discussions as the research assistants allocated each participant’s initials and transcribed the discussion of each participant verbatim. The socio-demographic questions for the focus group participants included gender, marital status, level of education attainment, and their source of livelihood (multi-response question). Because demographic characteristics can influence sharing within the group discussion, homogeneity with sufficient variation among members to support opposing viewpoints was established.

### 2.4 Data analysis

Statistical Package for the Social Sciences (SPSS) Version 21.0 was used to analyze the data. The chi-square test of association was used to establish the association between the independent and the dependent variables. For each item in the provision of social support questionnaire, the relationship to overall satisfaction in that domain of social support was assessed to establish whether it was significant. Because the sample sizes were small, Fisher’s exact test was preferred because it does not depend on any large-sample asymptotic distribution assumptions (Field & Miles, 2010). The results were reported with a 95% confidence interval (CI) and a *p-*value of 0.05*.* Descriptive statistics (frequencies, percentages) were also calculated and are presented in tables. Thematic analysis was used to analyze qualitative data. Thematic analysis is a technique for assessing qualitative data that involves looking through data to find, examine, and report recurring themes (Viji & Benedict, 2014). Kiger and Varpio (2020) contend that rather than choosing an easy-to-use method of analysis, deciding to utilize thematic analysis should be based on the objectives of the study itself. In this case, the focus group template of the key themes that were developed from the questionnaire study was checked against the focus group transcripts. Verbatim quotations from each of the domains were chosen to complement the quantitative data that represented the main themes that emerged from the discussions. To ascertain the trustworthiness of FGDs, the researcher and two research assistants engaged in transcribing and categorizing the themes. Final agreement on the themes for each domain of social support was reached by comparing the three researchers’ independently drawn theme categories. The data were triangulated across the four sub-counties by conducting one FGD in each. Verbatim quotations that were selected also reflected the main themes that emerged from the discussions, and there was prolonged engagement with the transcriptions as well as persistent observation of the emerging themes. According to Guion et al. (2011) triangulation establishes the validity of qualitative studies and, in this case, demonstrated that the emerging themes were trustworthy and plausible.

### 2.5 Ethical approval

Study participants were informed about the study by the researcher and research assistants. They were notified about the procedure to be followed and that participation was voluntary. They were made aware that they could withdraw from the study at any time and that the information they provided would be used for academic purposes only. The approval to conduct the study was granted by the Kenyatta University Graduate School and permitted by the Kenyatta University Ethical Review Committee (KU-ERC), approval number PKU/2235/11379, and the National Commission for Science, Technology, and Innovation (NACOSTI) [Permit number NACOSTI/P/21/11012.

## 3 Results

### 3.1 Socio-demographic characteristics

Most respondents were women (234, 59.1%) aged between 60 and 69 years. Of which, 200 (50.5%) were married, 228 (57.6%) had attained a primary level of education, 165 (41.7%) earned their livelihood from farming, and 224 (57.8) reported on average a monthly income of KES 1,001–5,000 (equivalent to USD 10–50). Results are shown in Table 1.

**TABLE 1:** Demographic profiles of FGD participants.

Variable	Categories	*n *= 369	(%)	*n* = 32	(%)
Gender	Male	162	40.9	14	47.3
	Female	234	59.1	18	56.3
Age	60–69	200	50.5	18	56.3
	70–79	110	27.8	12	37.5
	80–89	63	15.9	1	3.1
	90–99	17	4.3	1	3.1
	100+	6	1.5	0	0
Marital Status	Single	13	3.3	2	6.3
	Married	228	57.6	15	46.9
	Divorced	3	0.8	1	3.1
	Separated	9	2.3	1	3.1
	Widowed	143	36.1	13	40.6
Level of education	No formal education	142	35.9	6	18.7
	Primary	165	41.7	14	43.8
	Secondary	74	18.7	7	21.9
	Tertiary	15	3.8	5	15.6
Source of livelihood	Farming	284	57.8%	25	43.9
	Older persons cash transfer (OPCT)	94	19.1%	6	10.5
	Pension	31	6.3%	0	0
	Business	31	6.3%	0	0
	Casual labor	25	5.1%	2	8.8
	Support by children and other kin	18	3.7%	22	38.6
	Employment	8	1.6%	2	8.8
Average monthly Ksh	Below 1,000	99	25.0		
	1,001–5,000	227	57.3		
	5,001–10,000	25	6.3		
	Over 10,000	45	11.4		

### 3.2 Social support and social well-being

#### 3.2.1 Instrumental social support

The results show that most respondents provided social support within each item of instrumental social support and were satisfied with the social support they provided to CNMs. The calculated chi-square statistic using Fisher’s exact test demonstrated a highly statistically significant association between the instrumental social support variables and satisfaction with provided social support [satisfaction with lending money (χ^2^ = 6.05, *p* < 0.014)], satisfaction with providing care to CNM in sickness (χ^2^ = 13.26, *p* < 0.000), satisfaction with providing help with chores/errands (χ^2^ = 4.48, *p* < 0.034), satisfaction with lending household tools/items (χ^2^ = 8.90 *p* < 0.017)]. Results are shown in Table 2.

**TABLE 2:** Cross-tabulation of social support provided and satisfaction with provided instrumental support.

Support provided	Responses	Satisfaction with provided instrumental support				χ^2^	df	*p*-value
		Satisfied %		Dissatisfied %				
Lend money	Yes	281 (97.2%)	74.1%	8 (2.8%)	47.1%	6.05	1	0.014 e*
	No	98 (91.6%)	25.9%	9 (8.4%)	52.9%			
Care in sickness	Yes	325 (97.3%)	85.8%	9 (2.7%)	52.9%	13.26	1	0.000 e*
	No	54 (87.1%)	14.7%	8 (12.9%)	47.1%			
Help with household chores	Yes	287 (97.0%)	74.7%	9 (3.0%)	52.9%	4.475	1	0.034 e*
	No	92 (92.0%)	25.3%	8 (8.0%)	47.1%			
Lending household items/tools	Yes	358 (96.5%)	94.5%	13 (3.5%)	76.5%	8.901	1	0.017 e*
	No	21 (84.0%)	5.5%	4 (16.0%)	23.5%			

*Fisher’s exact test.

Participants in the focus group discussions agreed with the study findings that providing instrumental social support is pervasive among older people. They further alluded that providing for others is a good thing to do as long as it is within a person’s ability to provide. The narrative below demonstrates that providing instrumental social support elicited positive effects on older peoples’ social well-being:

My son was having a very hard time paying rent in Nairobi because of COVID-19 lockdown, I gave my son money to rent a cheaper house and felt good helping him (JM, 68-year-old male FGD participant).
I left my watchman job to attend to my sick brother because he is my brother and needed me (AG, 67-year-old male FGD participant).
I help in cleaning utensils because I can’t help in the farms and am happy am useful (JK, 84-year-old female).
I feel good when I help the people close to me with chairs and sufurias (cooking pot) because I know that they have sought my assistance since they don’t have the items (FK, 75-year-old female participant).

These narratives demonstrate that older people felt useful when they were able to meet the needs of their CNM. Therefore, providing instrumental support was satisfying for older people because it elicited a feeling of being useful to others when they met the needs of CNMs.

#### 3.2.2 Emotional social support

The results show that most respondents provided social support within each item of emotional social support and were satisfied with the social support that they provided to CNMs. The smallest expected frequency in the cross-tabulation Table 3 did not exceed 5, and the assumption of chi-square was not met. Fisher’s exact test (Fisher, 1922) was used to compute the exact probability of the chi-square statistic. The association between emotional support provided and satisfaction with providing emotional support was highly significant [satisfied cheering up or helping CNM feel better (χ^2^ = 32.54, *p* < 0.000), satisfaction with showing interest in their personal life (χ^2^ = 40.44, *p* < 0.000), satisfied doing or saying things that were kind or considerate (χ^2^ = 67.41, *p* < 0.000), satisfied helping CNM to solve their problems (χ^2^ = 41.83, *p* < 0.000)]. The results are shown in Table 3.

**TABLE 3:** Cross-tabulation of social support provided and satisfaction with provided emotional support.

Support provided	Responses	Satisfaction with provided emotional support				χ^2^	df	*p*-value
		Satisfied %		Dissatisfied %				
Cheered CNM up	Yes	351 (97.8%)	92.4	8 (2.2%)	50.0	32.538	1	0.000*
	No	29 (78.4%)	76	8 (21.6%)	50.0			
Showed interest in the personal life of CNM	Yes	350 (98.0%)	92.1	7 (2.0%)	43.8	40.435	1	0.000*
	No	30 (76.9%)	7.9	9 (23.1%)	56.3			
Did or said things that were kind or considerate	Yes	374 (97.4%)	98.4	10 (2.6%)	62.5	67.419	1	0.000*
	No	6 (50.0%)	1.6	6 (50.0%)	37.5			
Trusted CNM to solve your problems	Yes	351 (98.0%)	92.4	7 (2.0%)	43.8	41.834	1	0.000*
	No	29 (96.0%)	7.6	9 (4.0%)	56.3			

*Fisher’s exact test.

These findings were congruent with the voices of the FGD participants, who reported that they provide emotional support to CNMs and found fulfillment in this activity. This support is meant to help CNMs when they face difficulties, as explained in the excerpts below:

Out of my four children, three of them lost their jobs due to the COVID-19 pandemic. I cheered them up by reminding them of where we have come from and also sent them maize and beans from the village and encouraged them not to give up despite the hard times they were going through (DK, a 73-year-old FGD male participant).
My son had a fight with the wife and chased her away. She went to town and got a house for herself and the kids. I knew my son was in the wrong, and he was not listening to my scolding. I left my son in the village and joined his estranged wife in town and stayed with her until my son accepted her back. She is a good wife and exactly the person my son needs. I feel good they reconciled even if it compelled me to intrude in their personal lives (SK, a 69-year-old female participant).
I am always happy when my children are happy. I celebrate when they celebrate and thank God for them. Anytime they are unhappy, it affects me too (MK, a 70-year-old male participant).
I am the treasurer of the community self-help group. My son needed some money and borrowed it from me. I took some from the kitty and trusted my son to refund on time before our next community meeting. He refunded the money on time, and I was very proud of him for being worthy of my trust (AN, a 68-year-old male participant).

These voices from the FGD participants imply that older people go to great lengths to provide help to their CNMs. This is because when CNMs are facing challenges, the older people are also affected and thus put effort in helping the CNM return to a state that can potentially elicit positive emotions. When they succeed at it, their own social well-being is enhanced. Providing the emotional support was satisfying to older people by increasing their social connection and engagement with CNMs.

#### 3.2.3 Informational social support

The association between information support and satisfaction with providing information support was computed. The expected frequencies in the distribution were not greater than 5, and so the chi-square assumption was not met. Fisher’s exact test, a precise test that is suitable when samples sizes are small (Kim, 2017), was used, and it demonstrated significance between the variables giving helpful advice when needed to make important decision and satisfaction providing information support (χ^2^ = 73.02, *p* < 0.000), satisfaction agreeing with CNM’s actions or thoughts (χ^2^ = 41.55, *p* < 0.000), gave information to understand an issue (χ^2^ = 49.62, *p* < 0.000), gave CNM feedback on an action they wanted to take (χ^2^ = 39.01, *p* < 0.000). Results are shown in Table 4.

**TABLE 4:** Cross-tabulation of social support provided and satisfaction with provided informational support.

Support provided	Responses	Satisfaction with provided informational support				χ^2^	df	*p*-value
		Satisfied %		Dissatisfied %				
Offered helpful advice	Yes	358 (98.9%)	93.7	4 (1.1%)	28.6	73.025	1	0.000*
	No	24 (70.6%)	6.3	10 (29.4)	71.4			
Agreed with CNM’s thoughts/actions	Yes	347 (98.6%)	90.8	5 (1.4%)	35.7	41.549	1	0.000*
	No	35 (79.5%)	9.2	9 (20.5%)	64.3			
Gave CNM information to understand an issue	Yes	337 (99.1%)	88.2	3 (0.9%)	21.4	49.620	1	0.000*
	No	45 (80.4%)	11.8	11 (19.6%)	78.6			
Gave CNM feedback on an action they wanted to take	Yes	326 (99.1%)	85.6	3 (0.9%)	21.4	39.013	1	0.000*
	No	56 (83.6%)	14.4	11 (16.4%)	78.6			

*Fisher’s exact test.

The voices of FGD participants echo the findings from the quantitative analysis.

My neighbor has been quarreling with his neighbor about a land boundary and wanted to take the case to court. I suggested to him that the case could be solved at the clan level instead of going to court. He heard my advice, and the issue has been resolved (AW, 72-year-old male participant).
My neighbor had kept a man (co-habit), and the neighbors thought her actions were not right and the man was not good for her, but I saw him as a good man and agreed with her action to stay with him. I told her that she made a good decision to think about herself for once since her husband died ten years ago (AM, 72-year-old female participant).
My grandchildren have been raised in Nairobi and do not understand much about our Kamba culture. I explained to them when they visited about our clan, which is called *Anzauni*, about how hardworking we are. It felt good sharing about our clan with the children (AP 78-year-old male participant).
My brother came to me because he had problems paying school fees for his son who was to join university. He wanted to sell a portion of land that our parents left me to oversee. I did not have a response at the time and told him I would think about it. A few days later, I went to him and gave him reasons why we cannot sell that portion of land. I suggested ways we could raise the money without having to dispose our only prime land. My brother’s patience and willingness to look for alternative ways showed how much he respects me (SM, a 68-year-old male participant).

From these voices, it is evident that an effect is generated that has the potential to influence social well-being. Older people weigh in on whether the information support they provide will benefit the CNM. In addition, they feel knowledgeable and needed by the CNM, which benefits their social well-being. Actively offering information support to others was satisfying to older people by making them part and parcel of the decision-making process. Additionally, it increased communication between them and their CNM and gave them a sense of resourcefulness.

In summary, the study shows that each dimension of social support provided by older people was significantly associated with social well-being, as measured by participants’ reports of their satisfaction with that dimension. However, emotional and informational support had a stronger association than instrumental support.

## 4 Discussion

### 4.1 Instrumental social support

Social support is a multi-dimensional construct that Schaefer, Coyne, and Lazarus (1981) classified into three types: tangible/instrumental, informational, and emotional. Instrumental support includes doing things for others (DeHoff et al., 2016) and offering goods and services (Shakespeare-Finch & Obst, 2011; Southwick et al., 2016) that help solve practical problems. Generally, a statistically significant (*p* < 0.05) association was reported between providing instrumental social support and social well-being. The high significance recorded between the two variables could be attributed to the expectation that such support will be availed to older people at a later date. According to Chitaka (2017), the expectations of older people that the support they offered can be reciprocated when they need it is satisfying.

Generally, the significant finding that providing instrumental social support was associated with social well-being has been observed in the literature. Providing instrumental social support was associated with higher levels of life satisfaction (Brown et al., 2003) and greater life satisfaction among rural Taiwanese older adults (Ku et al., 2013). However, in a study on contributory behaviors and life satisfaction among older Chinese adults, Liu et al. (2019) found that providing instrumental social support and satisfaction with life were unrelated.

In the first instrumental item [lending money and satisfaction with lending money (χ^2^ = 6.05, *p* < 0.014)], satisfaction was attributed to the feelings of being useful to CNMs and the assurance of support during their own time of need. Older people have support expectations from children and grandchildren that they assist, in the present or past, in their time of need (Schatz & Ogunmefun, 2007; Chitaka, 2017). These findings resonate with a longitudinal study in Ireland that found that older people who provide financial support to CNMs have the highest quality of life compared to those who only receive support (Ward & McGarrigle, 2017). Similarly, spending money on others was found to produce positive effect in a study of 136 higher- and lower-income countries around the world (Aknin et al., 2013). The results are reinforced by the Blau (1968) tenet, which stresses that social actors engage in activities that entail some costs in order to obtain desired goals.

The second instrumental item [providing care in sickness and satisfaction with providing instrumental support (χ^2^ = 13.26, *p* < 0.000)] was also significantly associated with satisfaction with providing instrumental support. This significant finding was attributed to the unintentionality of being/getting sick as well as how providing care perpetuates life and survival. According to Inagaki and Orehek (2017), caring for others is not only a good thing to do but also necessary for the survival of our species. However, these findings are not in agreement with Milne et al. (2014), who found that the likelihood of older people providing care to sick CNMs is high and has the potential to affect their own health problems, which is not satisfying.

The significant results reported on the third item of the instrumental social support investigated [helping with household chores and satisfaction with providing instrumental support (χ^2^ = 4.48, *p* < 0.034)] could be attributed to this activity’s effect of keeping older people busy and active. According to Koblinsky et al. (2021), household chores may include activities such as cleaning, tidying, dusting, and home maintenance work like yard work and home repairs. These household chores keep older people active and serve as a low-risk form of beneficial exercise (Ward & McGarrigle, 2017; Koblinsky et al., 2021). The findings of this study corroborate those of Adjei and Brand (2018), which showed that older people were satisfied with their household chores because those chores were beneficial to their health and well-being. Similarly, the study by Crisp and Robinson (2010) also revealed that household chores enhanced the social well-being of older people and their relationships with CNMs.

The significant (*p* < 0.05) association between lending household tools/items and satisfaction with providing instrumental support (χ^2^ = 8.90 *p* < 0.017) was attributed to the feelings of being helpful to others and meeting a need. Lending household tools/items is a form of assistance in social relationships that serves as a resource to meet needs (Amurwon et al., 2017). This study’s results conform with Wang et al.’s (2019) findings that social reciprocity takes place in close relationships like those of CNMs and older people, which eases access to what one has and the other does not in a time of need and is thus beneficial to social well-being.

### 4.2 Emotional social support

Emotional social support includes being there for someone (DeHoff et al., 2016) and behavior that fosters and expresses feelings of being loved, respected, and cared for (Shakespeare-Finch & Obst, 2011; Southwick et al., 2016). The first item of emotional social support [cheering up or helping a CNM feel better and satisfaction with providing emotional support (χ^2^ = 32.54, *p* < 0.000)] was statistically significant. This was associated to the bond shared between the older person and a CNM as well as the opportunity to share knowledge and experience gained over the years. This finding agrees with Carstensen, Freedman, and Larson (2016), who noted that older people are in a unique position to serve as supporters and guides to CNMs, which allows the older people to experience fulfillment and purpose in their own lives. Similarly, in Kenya, Kimamo and Kariuki (2018) noted that older people often cheer their CNM, especially during major public holidays, by slaughtering chicken, sheep, or goats and joyfully sharing a meal with CNMs, while at the same time enquiring how each is doing in their personal lives. It is satisfying for older people when their CNMs are all cheerful in their home.

Showing interest in the personal life of a CNM was also significantly associated with satisfaction with providing emotional social support (χ^2^ = 40.44, *p* < 0.000). This significant association was attributed to CNMs and their personal concerns being important to older people. According to Vaillant (2012), older people are motivated to get involved in the lives of others. This interest in others is not limited to some aspects of life but to their great desire to lavish time, affection, information, concern, and resources on others (Erikson, 1994).

These results resonate with Amati et al.’s (2018) findings that older people provide CNMs with emotional support by showing interest in their personal or family matters, serving as a resource pool to help the CNMs solve their problems. The older people find it satisfying to be part of the solution. The findings of this study, however, do not concur with those of Juma, Okeyo, and Kidenda (2004), which showed that showing interest in the life of CNMs, especially those under their care like orphans, cause older people to worry about who will continue providing care when they die.

The third emotional item was doing or saying things that were kind or considerate and was significantly associated with satisfaction with providing emotional social support (χ^2^ = 67.41, *p* < 0.000). This significance was attributed to the effect generated when something kind was said to a CNM. When the CNM experienced pleasant emotions because of something the older person said or did, it resultantly produced a positive effect in that older person. Oerlemans, Bakker, and Veenhoven (2011) stated that people experience happiness as a pleasant and somewhat stimulated emotional state in their daily lives. The kind and considerate words or deeds from older people to CNMs stimulated pleasant emotions, which elicited satisfaction. These results are in agreement with a South African study by Makiwane (2010)thatshowed that older people are great encouragers who are happy about the success of the CNM and also offer support when things are not working well. Because the older people have amassed much experience, they can offer hope and wisdom to calm or cheer CNMs.

Lastly, trusting CNMs to solve their problems was significantly associated with satisfaction with providing social support (χ^2^ = 41.83, *p* < 0.000). This was attributed to the presence of a trustworthy person and the relief obtained when a viable solution to an issue was provided by a CNM. The presence of trustworthy CNMs whom older people can confide in provides access to social support that addresses older people’s problems (Storchi, 2017). These findings contradict those of the Clough et al. (2007) study, which reported that older people expressed concerns about not knowing whom to trust to solve their problems. He argued that older people fear for their safety and lives when they do not know or have someone they can trust to solve their problems. They face challenges and lack trustworthy people, which creates worry that affects their social well-being.

Generally, the highly significant association observed in the four items of emotional support and social well-being was attributed to the presence of CNMs who have a close relationship with the older person. According to Thomas, Liu, and Umberson (2017), closeness in family ties, particularly to adult children who give social support to their elderly parents, can have a major impact on well-being. Providing emotional support has been reported in prior studies as significantly associated with reduced stress (Shakespeare-Finch, & Obst, 2011), beneficial for the life satisfaction of aging parents (Silverstein, Cong, and Li, 2006) and has the potential to enhance satisfaction with life for older people regardless of gender (Liu et al., 2019). Similarly, Morelli et al. (2015) also observed that emotionally supportive individuals also report higher levels of satisfaction.

### 4.3 Informational social support

Informational social support is sharing knowledge and resources (DeHoff et al., 2016), provision of advice and guidance (Lu et al., 2016; Southwick et al., 2016), or advice regarding the environment (Shakespeare-Finch & Obst, 2011), which is intended to help individuals accomplish a task or cope with a difficult life situation. The results observed in the four items studied are significantly associated with satisfaction with providing information support.

In the first item, providing helpful advice when needed to make an important decision was significantly associated with the older person’s satisfaction with providing information support (χ^2^ = 73.02, *p* < 0.000). This was attributed to the feeling of being knowledgeable on a matter and contributing to a CNM’s ability to make sound decisions. According to Carstensen et al. (2016), older people possess an almost mystical ability to assist CNMs to develop capabilities to deal with daily concerns. The authors argued that older people tend to rely on past experiences, which are directly linked to their emotions, temporary and permanent goals, and desire to give back. These findings are in tandem with the Michel et al. (2019) study on the roles of a grandmother in African societies. They concluded that older people provide invaluable and unbiased advice to every family member and are satisfied taking the role of an adviser. Similarly, Crisp and Robinson (2010) found that older people offer useful advice to CNMs, especially on navigating life’s challenges, which makes them feel good about themselves.

In the second item, agreeing with a CNM’s actions or thoughts was significantly associated with satisfaction with providing emotional support (χ^2^ = 41.55, *p* < 0.000). The significant result was associated with the CNM’s decision to act according to the older person’s expectations. Otherwise, the older people could not accept the CNM’s decision to act contrary to what they had strongly suggested and advised. According to Noftle and Fleeson (2010), older adults are significantly more agreeable than younger adults. This is because they possess higher emotional control (Charles & Carstensen, 2007) to weigh the costs and benefits of the thoughts or actions presented to them, which corroborates this study.

Older people gave their CNM information to understand an issue, which was significantly associated with satisfaction with providing informational support (χ^2^ = 49.62, *p* < 0.000). This was attributed to the positive effects elicited when older people gave a CNM information to understand an issue, which was satisfying to them. Annear et al. (2017) indicated that older people have wisdom, values, and skills that can benefit the younger generation. They possess a wide array of information, knowledge, and expertise that they pass along to CNMs (Michel et al., 2019). These findings are in agreement with those of Chadha (1999), who found that CNMs consult older people on a majority of life’s major issues, which makes older people feel useful and valued.

inally, giving feedback to a CNM on an action that they wanted to take was significantly related to satisfaction with providing information support (χ^2^ = 39.913, *p* < 0.05). This was attributed to the feelings of being valued arising from being consulted on a matter and being given time to think things through and offer feedback. According to Storchi (2017), when contacted about a subject, older people contribute feedback to a CNM because they feel valued and significant. Therefore, they take time to find the best possible response in a situation because they are strategic communicators who want to make a difference in the lives of others (Carstensen et al., 2016). The findings of this study are in agreement with Carstensen et al. (2016), who reported that before giving feedback on any issue, older people tend to rely on their past experiences, which are directly linked to their emotions, short- and long-term goals, and desire to give back. Being in a position to provide feedback when requested contributes to their satisfaction with life.

Overall, providing information support, as evidenced in the four items, significantly influenced satisfaction with providing information support. Having gone through different challenges and experiences, older people are able to understand the informational needs of a CNM and offer useful information (Heaney & Israel, 2008). This finding concurs with other studies that found that providing information support to others is linked to better health outcomes and well-being (Brown et al., 2003). Generally, providing social support in the three dimensions requires both cognitive judgments of satisfaction with social support and affective appraisals of moods and emotions about that support (Tov & Diener, 2013). The older people had to consider the help they offered to CNMs and how doing so affected their moods and emotions.

The findings of this study contribute significantly to the limited research evidence on the relationship between social support in the three categories and social well-being among older individuals in Kenya. The strengths of this study lie in its use of a randomly selected sample of older people, a high response rate, and detailed information on provided social support. The use of mixed methods—quantitative data from the questionnaire and qualitative data from focus group discussions—allowed the study to examine the association between social support and social well-being and obtain a deeper understanding through narratives, revealing subjective appraisals of providing support. The potential in the data lies in its scope and detail of the social support variables provided across the three domains.

The main shortcoming of this study was that the data are cross-sectional, and thus, the causal ordering of providing social support and social well-being is precluded. In future studies, data from longitudinal studies would be necessary to provide a temporal order of the measured variable. Future studies could also examine the resources available to older people providing social support to ascertain whether there are variations in social well-being among those with greater resources compared to those with fewer resources.

## Data Availability

The original contributions presented in the study are included in the article/Supplementary Material; further inquiries can be directed to the corresponding author.
